# Structural, Mechanical and Flammability Characterization of Crosslinked Talc Composites with Different Particle Sizes

**DOI:** 10.3390/ma15134492

**Published:** 2022-06-25

**Authors:** Beata Podkościelna, Tomasz Klepka, Przemysław Podkościelny, Anita Bocho-Janiszewska, Tomasz Wasilewski, Łukasz Klapiszewski

**Affiliations:** 1 Department of Polymer Chemistry, Institute of Chemical Science, Faculty of Chemistry, Maria Curie-Skłodowska University, M. Curie-Skłodowska Sq. 3, PL-20031 Lublin, Poland; 2 Department of Technology and Polymer Processing, Faculty of Mechanical Engineering, Lublin University of Technology, Nadbystrzycka 36, PL-20618 Lublin, Poland; t.klepka@pollub.pl; 3 Department of Theoretical Chemistry, Faculty of Chemistry, Maria Curie-Skłodowska University, M. Curie-Skłodowska Sq. 3, PL-20031 Lublin, Poland; przemyslaw.podkoscielny@poczta.umcs.lublin.pl; 4 Department of Industrial Chemistry, University of Technology and Humanities in Radom, Chrobrego 27, PL-26600 Radom, Poland; a.janiszewska@uthrad.pl (A.B.-J.); tomasz.wasilewski@uthrad.pl (T.W.); 5 Institute of Chemical Technology and Engineering, Faculty of Chemical Technology, Poznan University of Technology, Berdychowo 4, PL-60965 Poznan, Poland

**Keywords:** talc, methacrylate, N-vinyl-2-pyrrolidone, crosslinked composites, thermal and mechanical properties, flammability tests

## Abstract

The influence of filler particle size on selected physicochemical and functional properties of polymer composites was analyzed. The following test was carried out for the system: the bisphenol A glycerolate (1 glycerol/phenol) di-methacrylate (BPA.DM) was subjected to UV-polymerization in bulk with N-vinyl-2-pyrrolidone (NVP) as a polymer matrix and talc with particle sizes ranging from ≤8 to 710 µm as a non-toxic and cheap mineral filler. An effective method of preparing cross-linked polymeric composites with talc was developed. The obtained samples were subjected to structural analysis and the thermal, mechanical and flammability properties were assessed. It has been empirically confirmed that the talc particles are incorporated into the composite structure. However, with increasing particle size, the composite heterogeneity increases. In the case of the developed method of sample production, homogeneous systems were obtained for particles in the ≤8–250 µm range. The surface roughness of the samples correlates directly with the size of talc particles. The value of Young’s modulus during the axial stretching of samples decreases with the increasing size of talc particles. For the composites containing ≤15 and ≤35 µm talc particles, the highest values were obtained under bending conditions. There was no equivocal effect of particle size on the composites’ swelling in water. The addition of talc reduces the flame height and intensity slightly. The biggest difference was obtained for the composites containing relatively large talc particles. It was proved that the selected properties of polymer composites can be controlled depending on the size of the talc particles.

## 1. Introduction

Minerals are widely employed as fillers in the plastic industry. As the most commonly applied minerals one can include: calcium carbonate, clay, silica, talc, kaolin, mica, wollastonite and alumina trihydrate [1,2,3,4]. Besides the reduction in production costs, mineral fillers affect the final mechanical features of polymers or polymeric composites. They induce changes in the morphology of the polymeric chains and modify the volumetric properties of composites [5,6,7,8,9,10]. Additionally, the filler morphology, particle size, shape and distribution or crystalline degree have a major influence on the action, processing and properties of polymer composites [11,12,13].

The properties of polymer–inorganic composites are largely related to the interfacial adhesion between the polymeric matrix and the filler. The non-polar and chemically inert polyolefins (e.g., polyethylene—PE, polypropylene—PP) are the most common polymers. They do not interact with most inorganic fillers, which results in poor dispersion and weak interfacial adhesion. In consequence, reduced physical properties are observed [14,15]. To overcome the limitations associated with the preparation of non-polar composites with different fillers, various coupling agents such as phosphate silanes, titanium esters, aluminates and zirconates have been used [7,16].

Another way to enhance the compatibility of bulk polymers with inorganic fillers is by functionalization of polymer composites. The typical way to functionalize polyolefins is by grafting polar monomers, e.g., acrylic or methacrylic monomers or styrene, onto the polymer backbone. The functional polymeric compounds can interact strongly with fillers and polymeric chains. However, this technique has some limitations (e.g., crosslinking effect, degradation of polymeric chains or high degree of volatilization). A better effect can be achieved by using reactive polar monomers [17,18,19,20].

Talc (Mg_3_Si_4_O_10_(OH)_2_) is a clay mineral generally applied as an additive in polymeric composites, especially in the context of flame retardancy [21]. Wang et al. reported the methodology of composite fabrication based on polypropylene and talc fillers obtained by grinding pulverization. The properties of the materials made by the new method and by using pulverized talc methods were compared. Composite morphology studies show particle orientation and homogeneous dispersion of pulverized talc in the polypropylene matrix [22]. Wawrzyn et al. described four different inorganic additives, investigated for bisphenol A derivatives to improve flame retardancy. The pyrolysis, small flame response and fire behaviour of the blends were examined and the structure–property interactions were taken into consideration. Among the applied layered materials, talc was found to be a neutral additive while the modified montmorillonite improved decomposition [23]. Polymeric blends which are derivatives of bisphenol A polycarbonate with acrylonitrile/butadiene/styrene (PC/ABS) were investigated by Schartel et al. in terms of their fire behaviour, pyrolysis and flammability. It was established that the presence of talc had an impact on the pyrolysis and flammability results, since it contributed to a reduction of gas diffusion and increased the flow limit at small shear rates. Furthermore, the use of talc allowed enhancement of the protective properties of fire residues and also to partially suppress the flame inhibition and charring effect caused by bisphenol A bis(diphenyl phosphate) [24]. Qiu et al. studied the effects of talc size on structure and properties of composites based on poly(vinyl alcohol) (PVA). The results showed that reducing the talc particles increased the number of –OH groups at the edges of the talc layers, which improved the compatibility between talc and PVA. Additionally, the smaller talc particles were more uniformly dispersed in the PVA matrix [25]. The effect of particle size on the dimensional stability, morphology and mechanical properties of composites based on polycarbonate, poly(butylene terephthalate) and talc was investigated by DePolo and Barais [26]. It was found that the use of talc nanoparticles (instead of fine talc) brought about an increase in flexural and tensile strength, and a decrease in the density of talc composites. Compatibility of the polymer matrix and filler is a key issue from the point of view of the technology of producing composites. A properly selected system allows the elimination of additional substances from the structure of the composite responsible for reducing the interfacial tension. Moreover, proper compatibility eliminates the need to chemically modify the filler surface.

Talc is commonly used as an inexpensive filler for thermoplastic polymers but its application for bisphenol A derivatives has not been extensively studied. Due to many studies confirming the carcinogenic effect of bisphenol A, its use in food packaging has been restricted in a number of countries. On the other hand, its application in industrial chemistry has not diminished and it still finds many applications.

Bisphenol A derivatives (epoxy resins, acrylates) are widely used in powder paints, varnishes (chemical-resistant, insulating, electro-insulating), and adhesives for metals, glass, ceramics, thermosetting plastics. They are also found in the construction industry e.g., as sealing and flooring materials. Because bisphenol A products are largely employed in the direct human environment, the study of flammability and degradation of the prepared composites is interesting and purposeful [22].

Herein, the effective method for the preparation of cross-linked polymeric composites with different particle sizes of talc is presented and their thermal, mechanical and flammability properties are discussed in detail. Our goal is to show how the use of a raw material with different particle sizes affects the properties of composites. As a main crosslinking monomer the bisphenol A glycerolate (1 glycerol/phenol) di-methacrylate (BPA.DM) was used. The UV-polymerization of BPA.DM monomer with N-vinyl-2-pyrrolidone via the bulk polymerization technique is investigated. Talc with different particle sizes was used as a nontoxic and cheap filler. Various fractions of ground talc were applied in the research, and in scope covered particles with sizes from ≤8 to 710 µm. The influence of talc particle size on the quality and properties of the composites was evaluated. The spectroscopic studies of the obtained composites were characterized by means of the attenuated total reflection-Fourier transform infrared (ATR-FTIR) spectroscopy. The influence of the filler addition on the structural, mechanical and thermal properties was evaluated.

## 2. Materials and Methods

### 2.1. Materials

Bisphenol A glycerolate (1 glycerol/phenol) di-methacrylate (BPA.DM), N-vinyl-2-pyrrolidone (NVP) and 2,2-dimethoxy-2-phenylacetophenone (Irgacure 651, IQ) were purchased from Merck, Darmstadt, Germany. The talc particles were obtained from Elementis Minerals (East Windsor, NJ, USA). The following talc products were used: (i) Mondana 8 HT, (ii) Mondana 15 HT, (iii) Mondana 35 HT, (iv) Mondana 50 HT, (v) Mondana Scrub 250, (vi) Mondana Scrub 500 and (vii) Mondana Scrub 710. The values given next to the name mean that 98% of the talc particles are of an equal or smaller size (d98%) in µm. All chemicals were used as received.

### 2.2. Analytical Methods

Assessment of morphological properties was carried out using the FEI Quanta 250 FEG Scanning Electron Microscope (SEM) (FEI Inc., Eindhoven, The Netherlands). Untreated samples were fixed on the aluminum stubs using double-sided carbon tape and analyzed in the low vacuum mode with an atmospheric pressure of 130 Pa, avoiding typical coating of the artefacts. A dispersive X-ray (EDX) spectrometer (Octane Elect Silicon Drift Detector, EDAX, AMETEK Inc., Berwyn, PA, USA) was used for elemental identification and quantitative compositional analysis of the energy.

Visual inspection of solid composites was carried out using the optical microscope Morphology G3 (Malvern Panalytical, Malvern, UK).

A Bruker Tensor 27 FTIR spectrometer (Bruker Optics GmbH & Co. KG, Ettlingen, Germany) was used to record FTIR spectra based on the attenuated total reflectance (ATR) technique for samples in the form of thin films. All measurements were conducted at room temperature in the range of 4000–600 cm^−1^. The presented spectra are based on averaging 32 scans with a resolution of 4 cm^−1^ in the absorbance mode.

Netzsch Differential Scanning Calorimetry (DSC) 204 F1 Phoenix calorimeter (NETZSCH GmbH & Co. Holding KG, Selb, Germany) operating in a dynamic mode was employed to conduct the calorimetric measurements, using the following experimental set-up: a heating rate of 10 °C/min, from room temperature to a maximum of 550 °C, with nitrogen as the inert gas (30 mL/min). The mass of the samples was equal to approx. 10 mg and an empty aluminum crucible was used as a reference. The reported transitions came from the first heating scans.

An optical profilometer (Contour GT-K1, Veeco, Aschheim/Dornach Munich, Germany) was used to investigate the morphological images and surface roughness of the samples. This device is characterized by high accuracy in the size range from the sub-nanometer to 10 mm; nevertheless, the imaging was repeated 3 times for each sample in order to determine the average roughness (R_a_) parameter.

A Zwick7206/H04 durometer (Zwick Roell Group, Ulm, Germany), type D, was employed to carry out the Shore hardness tests (measurements were taken after 15 s at a temperature of 21 °C).

The equilibrium swelling in selected organic solvents and distilled water were used to determine the swelling coefficients (B) based on the following equation [27]:

(1) $$B=\frac{m_{s}-m_{d}}{m_{d}}\times 100\%$$

where *m_s_* is the mass after swelling and *m_d_* is the mass of the dry sample.

A Zwick/Roell Z010 universal tensile-testing machine (Ulm, Germany) was used to carry out strength tests for samples in the form of a pressed sheet. Uniaxial tensile strength tests were performed using 4 mm × 10 mm × 60 mm samples at a test speed of 50 mm/min and at 23 °C, in accordance with EN-ISO 527. The analysis of bending strength was conducted based on three-point bending tests, in accordance with EN-ISO 170 and ASTM D-790.

Subsequent testing of flammability was based on the horizontal burning test carried out in a device that included a combustion chamber, ventilation system and thermal imaging camera. The procedure is in accordance with the PN-EN 60695-11-10—method A. The samples were placed on a tripod and moved near a methane burner at an angle of 45°. After 10 s, the samples were left to burn freely for 30 s. The measurement included collection of thermal images of the samples (after every 5 s), measurement the total burning time and examining of the length of the sample after burning. The images of samples were collected using a V-20 thermo-vision camera model ER005-25 (Vigo System, Ożarów Mazowiecki, Poland), which operates in the temperature measurement range from −10 to 250 °C with a measurement accuracy of ±2 °C.

### 2.3. Synthesis of Composites

**Figure 1:**
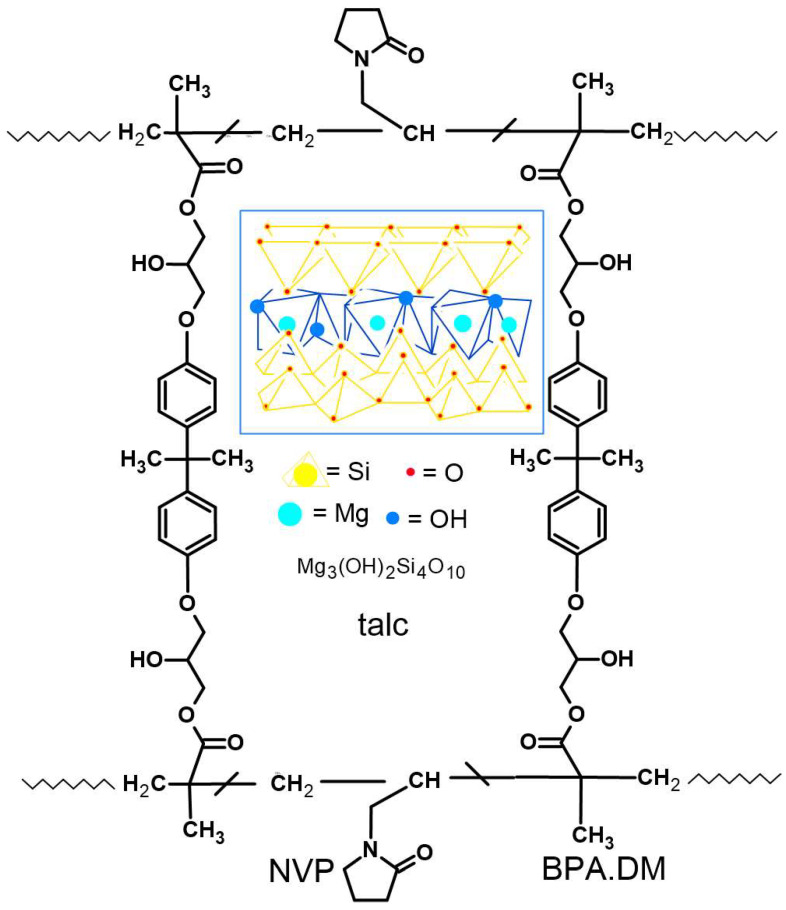
Assumed fragment of the composite structure.

In order to obtain the composites tested in this study, BPA.DM and NVP were introduced into a glass vessel at a ratio of 70:30 wt%. After subsequent mixing to ensure proper homogenization, the vessel was transferred to a heating chamber (60 °C) and the UV initiator (IQ) was added (at an amount equal to 2 wt% of the initial polymer mixture). An appropriate (20 wt%) amount of talc particles (8, 15, 35, 50, 250, 500 or 710 µm) was added in the last step. Then, the vessel was subjected to gentle stirring and placed in the heating chamber again (60 °C) for approx. 20 min. Afterwards, the mixture was poured into 100 mm × 80 mm glass moulds equipped with Teflon spacers. Finally, the mixture was exposed to UV light (15 min) inside the irradiation chamber with four mercury lamps of 40 W [28,29]. All the relevant parameters of the experimental set-up are presented in Table 1, whereas the assumed structure of the obtained composite is presented in Figure 1.

## 3. Results and Discussion

As a result of the UV polymerization reaction, eight composites, differing in particle size, of talc additive ranging from ≤8 to 710 µm were obtained (see Table 1). Raw material without any modification was used for the synthesis of compositions. The stated particle sizes shown in the talc data were provided by the producer, and the figure states the maximum particle size. In the case of six composites the material with the uniform filler dispersion was obtained. On the other hand, the talc particle sizes of ≤500 and ≤710 µm were found be too heavy, and talc falling to the bottom of the mold was observed. Therefore, the samples BPA.DM-NVP-0 to BPA.DM-NVP-5, uniform in their volume, were selected for thermal, swelling, profilometer, mechanical and flammability tests.

### 3.1. Visualization of the Studied Materials

**Figure 2:**
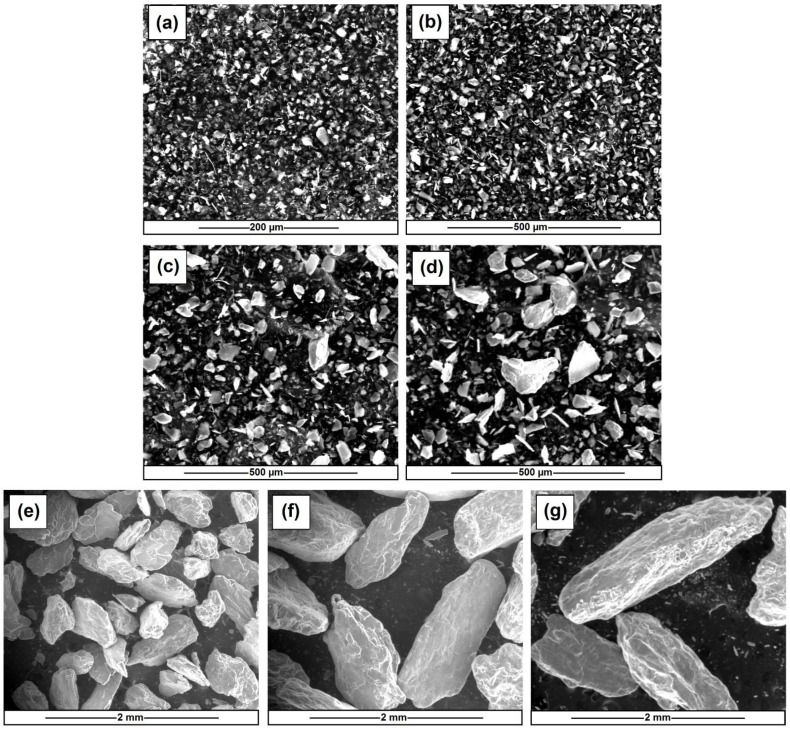
SEM images of talc: Mondana 8 HT (**a**), Mondana 15 HT (**b**), Mondana 35 HT (**c**), Mondana 50 HT (**d**), Mondana Scrub 250 (**e**), Mondana Scrub 500 (**f**) and Mondana Scrub 710 (**g**).

In Figure 2 the images of talc (with different sizes, used as a filler in the composites) by means of scanning electron microscope (SEM) analysis are presented. Due to the thickness of the samples, a better quality of polymerized composites image was obtained using an optical microscope.

**Figure 3:**
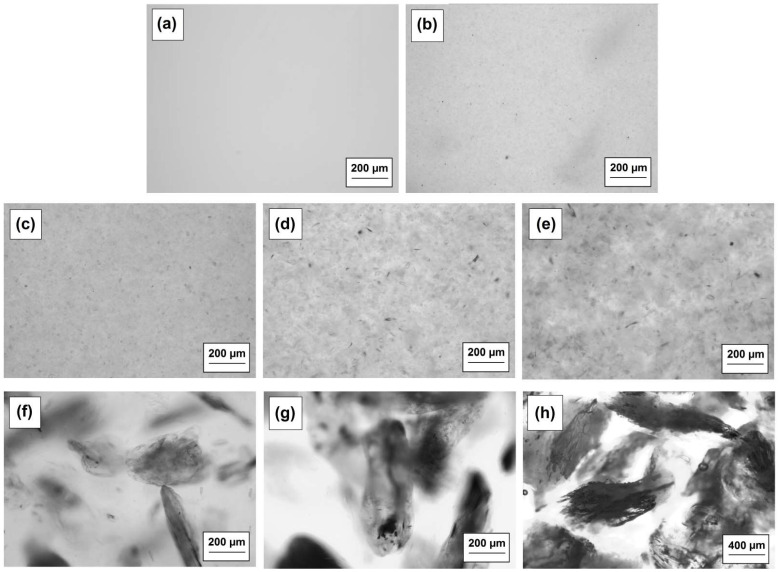
Photos of the composites made by the optical microscope: BPA.DM-NVP-0 (**a**), BPA.DM-NVP-1 (**b**), BPA.DM-NVP-2 (**c**), BPA.DM-NVP-3 (**d**), BPA.DM-NVP-4 (**e**), BPA.DM-NVP-5 (**f**), BPA.DM-NVP-6 (**g**) and BPA.DM-NVP-7 (**h**).

Figure 3 presents the photos of the fragmentary composite structure with different sizes of talc. Samples 2–5 (see Figure 3b–e) are characterized by a homogeneous structure. In contrast, the materials with the largest size of talc (≤250–710 µm) are characterized by clearly visible filler particles. In addition, in the case of samples 6–8 (see Figure 3f–h), the talc fragments are very large, and irregular distribution of the filler in the compositions is observed.

### 3.2. ATR/FTIR Spectra of the Obtained Composites

**Figure 4:**
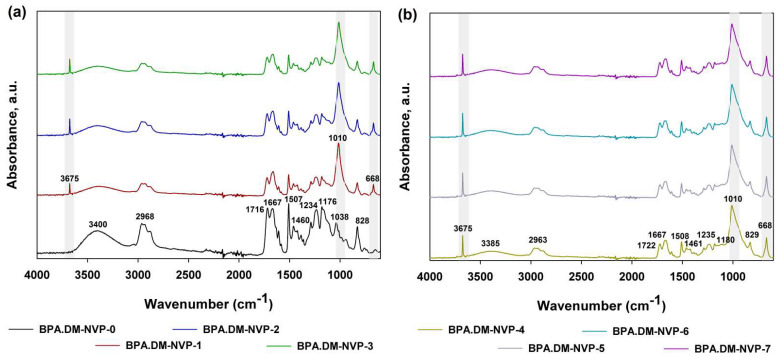
ATR/FTIR spectra of the prepared composites: BPA.DM-NVP-0–BPA.DM-NVP-3 (**a**) and BPA.DM-NVP-4–BPA.DM-NVP-7 (**b**).

The ATR/FT-IR spectra for the BPA.DM-NVP copolymers with various talc content are presented in Figure 4a,b. Comparison of spectra of the starting material and that of modified composites revealed the most notable changes (which are marked in Figure 4). The main differences are associated with the presence of a strong signal (at approx. 3400 cm^−1^) which corresponds to the –OH groups, bands assigned with the C–H stretching vibrations (asymmetric at approx. 2968 cm^−1^ and symmetric at 2850 cm^−1^) and bending vibrations of the –CH_3_ group (at 1460 and 1435 cm^−1^).

Furthermore, there are two distinct signals, which should be highlighted. The first is a sharp band (at 1716 cm^−1^) that can be attributed to the stretching and out-of-plane-bending vibrations of the carbonyl (C=O) group. The second (at 1667 cm^−1^) is associated with the N–C=O stretching vibration originating from the amide group (NVP). The presence of talc contributed to visible changes in the spectra, namely, the presence of two new signals (at 3675 and 668 cm^−1^). It is also worth noticing that a characteristic band (at 1010 cm^−1^) which can be attributed to the asymmetrical stretching vibrations of Si–O–C and Si–O–Si groups can be observed in the spectrum. The presence of this signal is a direct confirmation that talc has been incorporated into the composite structure.

### 3.3. SEM-EDX Analysis

**Figure 5:**
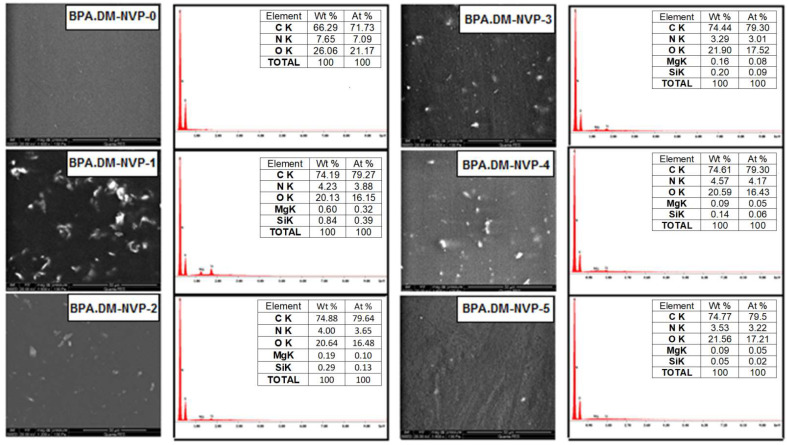
SEM images and SEM-EDX microanalysis of the samples.

SEM images of the sample surfaces and the corresponding results of the EDX microanalysis are presented in Figure 5.

The area of the tested surface was the same for all samples. The SEM images were obtained using the backscattered electron (BSE) mode. In this mode the images of the sample containing elements with a higher atomic number are mapped to the BSE images as brighter. Hence, the talc particles that contain Mg and Si are visible as bright spots in the picture. The presence of talc in the polymer structure is also confirmed by the EDX microanalysis. It can be seen that, apart from the elements C, N and O, Mg and Si are also present in the talc structure. The SEM images and the EDX analysis show that the talc particle size corresponds to the frequency of its appearance on the surface. The greatest number of talc particles is located in the surface layer of the polymer containing the smallest sized talc particles.

### 3.4. DSC Analysis

**Figure 6:**
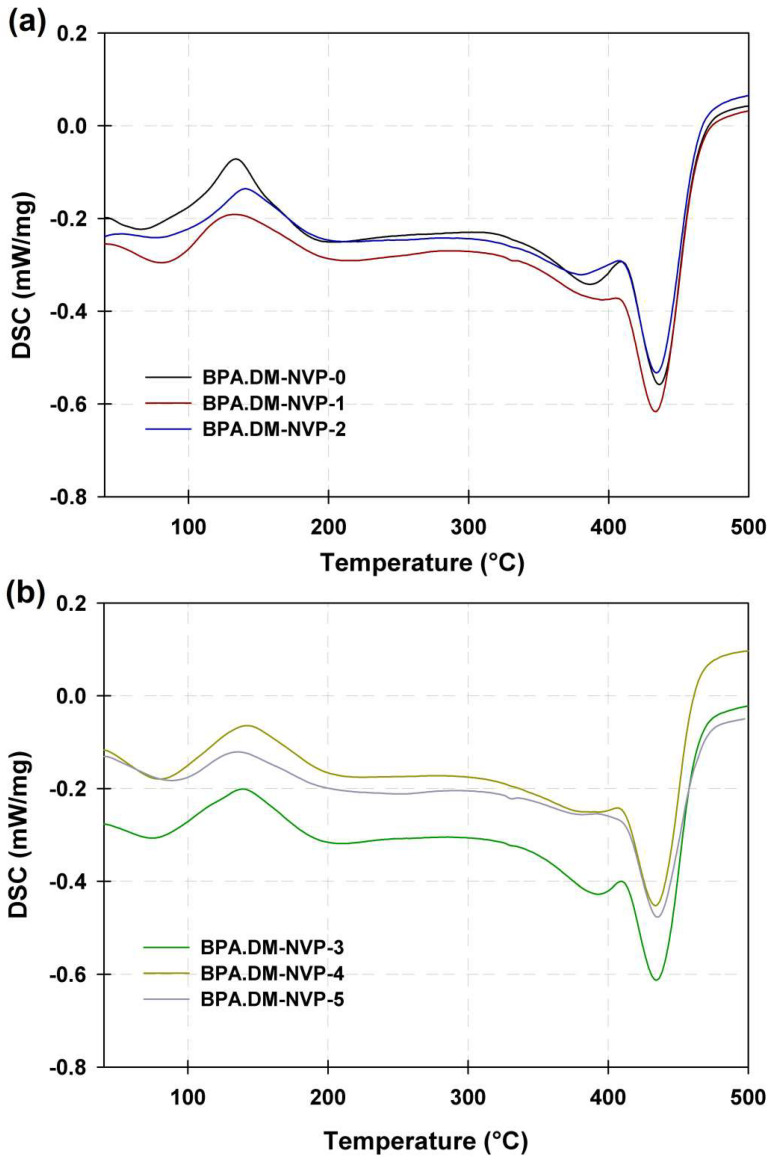
DSC curves of the polymer composites: BPA.DM-NVP-0–BPA.DM-NVP-2 (**a**) and BPA.DM-NVP-3–BPA.DM-NVP-5 (**b**).

In Figure 6 the curves for the samples in the range from 25 to 500 °C are visible.

As one can see for samples BPA.DM-NVP-0–BPA.DM-NVP-2 (see Figure 6a), two small endothermic effects are visible. The first at about 90 °C is associated with evaporation of unreacted NVP, and the second at about 390 °C corresponds to the degradation of the linear part of aliphatic NVP. On the analyzed curves, there is also a large endothermic effect in the range 431–442 °C associated with the total thermal degradation of the samples. For all composites at ca. 150 °C, the exothermic effect, probably related to the crosslinking reaction, is visible. For samples BPA.DM-NVP-3–BPA.DM-NVP-5 (see Figure 6b), two endothermic and one exothermic effects are noticeable. The main endothermic effect at about 445 °C was due to the total thermal degradation of the samples [29,30]. The addition of talc affects the thermal resistance of the obtained materials positively. The temperature of composites’ maximum degradation increases slightly (3–7 °C) but greater homogeneity of the samples is observed (decrease in the endothermic signal at 390 °C).

### 3.5. Swelling Studies in Water

The samples with talc particle sizes ranging from 8–250 µm were selected for the evaluation of physical and mechanical properties.

**Figure 7:**
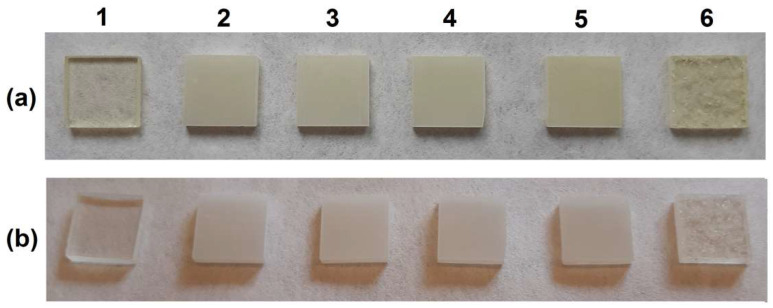
Photos of the samples after cutting (10 mm × 10 mm) for the swelling measurements: before swelling (**a**) and after swelling (**b**) in water (numbers of the sample according to Table 1).

In order to determine the swelling coefficients (B), samples of the tested material (approx. 0.26 g) were immersed in distilled water for a specific time period (two weeks, as well as with short-term contact i.e., after 15, 60 and 720 min). The corresponding swelling coefficients are listed in Table 2, whereas the initial and final state of the samples are presented in Figure 7.

Based on the obtained results, it can be concluded that the composites with talc are stable in water. The samples, as presented in Figure 7b, do not change their shape. Moreover, the changes on the composites’ surface are visible. After 2 weeks in the environment of water, their swelling coefficients are in the range of 4.2–5.3%. Due to the cross-linking structure the composites’ immersion in water does not result in their dissolution. However, these bonds do not inhibit swelling of the cross-linked composites.

### 3.6. Profilometer Analysis

**Figure 8:**
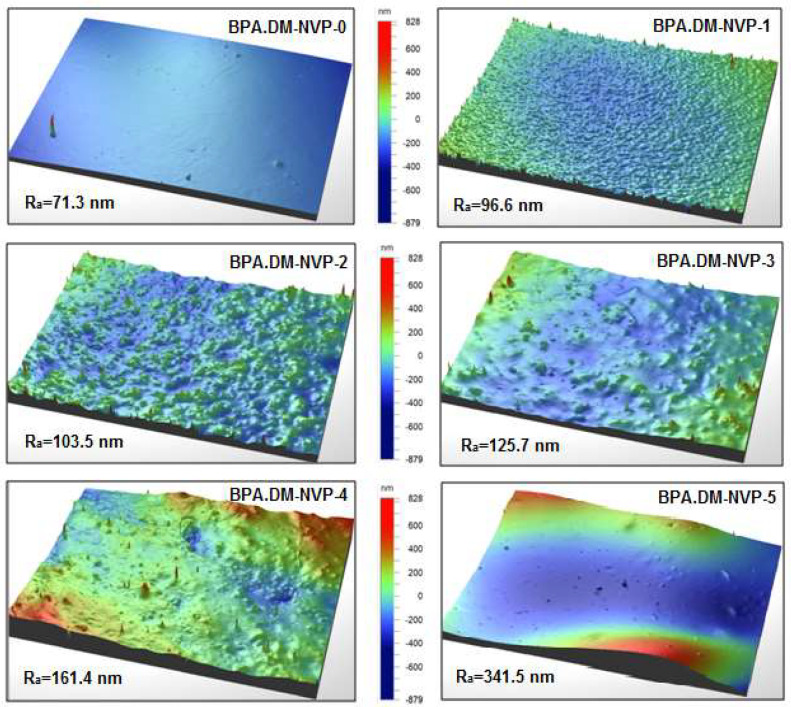
Surface images from the profilometer.

The profilometric analyses of the composites shows the significant changes in the surface of the studied materials. The analysis of the arithmetic average roughness R_a_ values confirms the significant differences in the surface structure of the tested material. An increase in roughness directly proportional to the talc size (increase R_a_ from 71.3 to 341.5 nm) is evident (see Figure 8).

### 3.7. Mechanical Properties of Composites

The investigations of mechanical properties showed how the filler size affects the strength properties of the tested samples depending on the direction of external excitations. The summary results of the mechanical properties tests are presented in Table 3.

**Figure 9:**
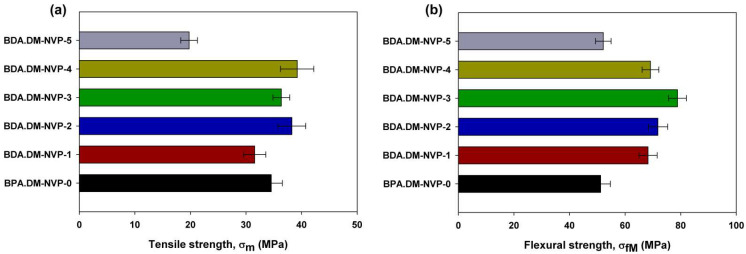
Stress diagram for various test specimens obtained during the uniaxial tension (**a**) and the bending test (**b**).

The values of stress determined by the uniaxial tension and bending tests as well as that of the Young’s modulus describe the elastic properties [31,32]. Depending on the structure of the composite and the type of external forcing during the tests under uniaxial tension, the value of Young’s modulus decreases proportionally to the increasing size of talc particles added into the polymer matrix. However, when tested under bending conditions (see Figure 9b) two clear maxima of the highest values were observed for the samples BPA.DM-NVP-2 and BPA.DM-NVP-3. During bending a complex stress state is induced in the tested sample. At the polymer–filler interface the external forces act with higher intensity in the case of the talc sizes in the range of 15 to 35 µm (BPA.DM-NVP-2 and BPA.DM-NVP-3 samples). For the smallest filler particles (8 µm), due to their aeration effect (e.g., when mixing the filler with monomers), there are weaker filler–polymer interactions in the polymer matrix. In the case of very large talc particles, despite the lack of aeration effect, the mechanical properties decrease along with weakening the polymer network at the mineral–polymer interface (BPA.DM-NVP-5).

**Figure 10:**
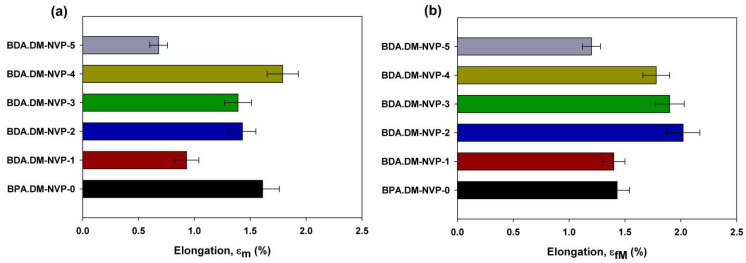
Elongation diagram during tensile (**a**) and bending (**b**) tests.

Analyzing the results of the deformation test, it is clear that in the complex state of stress that occurs during bending, the highest values (see Figure 10b) were obtained for the specimens BPA.DM-NVP-2 and BPA.DM-NVP-3. However, in the case of uniaxial stretching where the force acts only in one plane, the smallest elongation was observed for the smallest and the largest values of talc grains (see Figure 9a).

### 3.8. Flammability Tests

**Figure 11:**
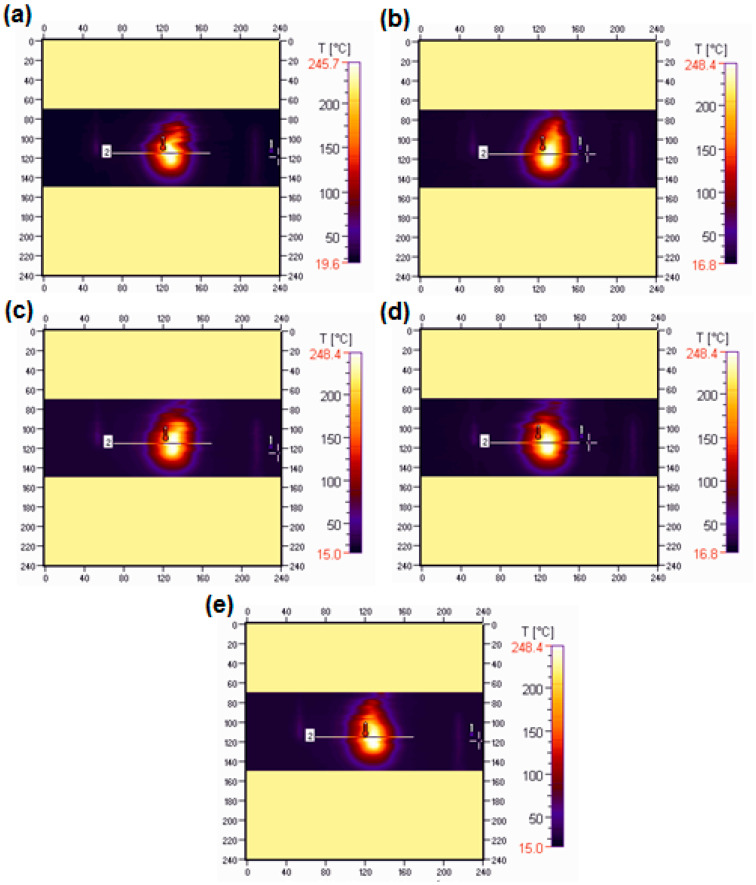
Thermograms of sample 1 (BPA.DM-NVP-0) recorded: 20 (**a**), 25 (**b**), 30 (**c**), 35 (**d**) and 40 (**e**) seconds after the ignition source application.

**Figure 12:**
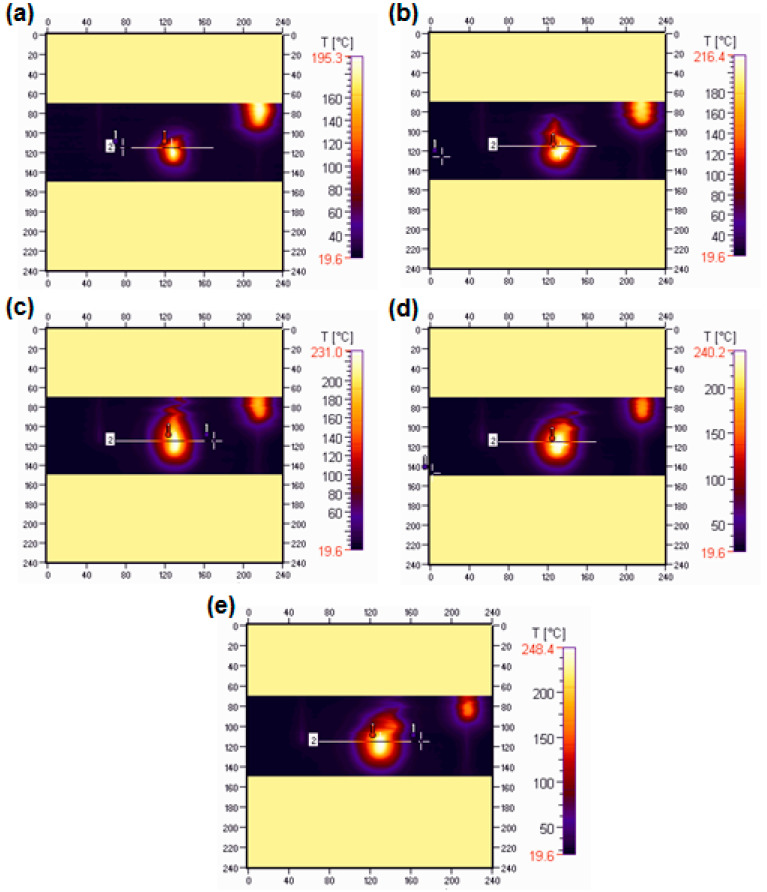
Thermograms of sample 6 (BPA.DM-NVP-5) recorded: 20 (**a**), 25 (**b**), 30 (**c**), 35 (**d**) and 40 (**e**) seconds after the ignition source application.

**Figure 13:**
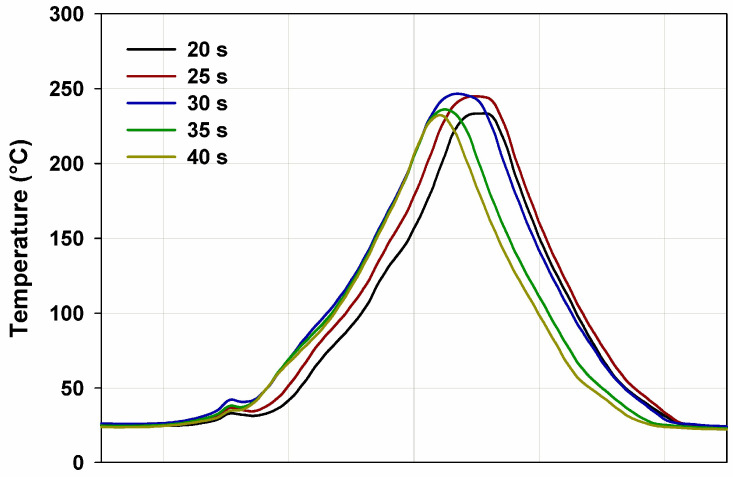
Temperature (T_1_) along the free burning sample 1 (BPA.DM-NVP-0).

**Figure 14:**
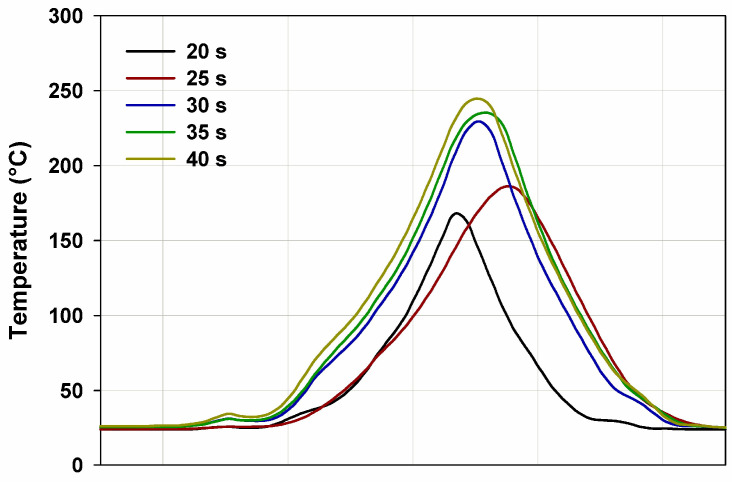
Temperature (T_1_) along the free burning sample 6 (BPA.DM-NVP-5).

The flammability research was carried out in a horizontal burning test. The samples were burning for 60 s (15 s over a burner and then self-burning for 45 s). All samples burned with a bright smokeless flame. In Figure 11 and Figure 12 the images of the selected samples 1 (BPA.DM-NVP-0) and 6 (BPA.DM-NVP-5), respectively, taken using a thermographic camera are visible. The results contained in Table 4 are shown for the temperature measuring range 20–500 °C. The graphs were made using a computer program Vigo System (Ożarów Mazowiecki, Poland). The analysis of the temperature field in the area of the burning sample includes the determination of the changes of the maximum temperature in the whole thermal image (area 1, T_1_) recorded at 20, 25, 30, 35 and 40 s after the application of the ignition source (see Figure 13 and Figure 14). The temperature along a straight line running through the longitudinal axis of the sample (area 2, T_2_) was also measured (see Table 4).

For all samples with talc, reduction of the initial burning temperature recorded with the camera is observed. The average temperature for sample 1 (BPA.DM-NVP-0) is 248 °C, while for sample 6 (BPA.DM-NVP-5) this is 223 °C. The addition of talc decreases the flame temperature by about 10%.

**Figure 15:**
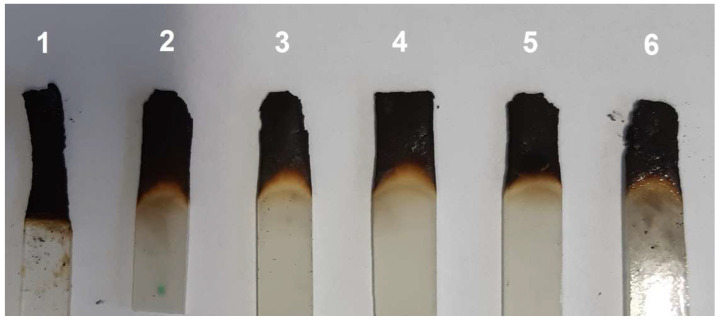
Samples after the burning test: sample 1—BPA.DM-NVP-0, sample 2—BPA.DM-NVP-1, sample 3—BPA.DM-NVP-2, sample 4—BPA.DM-NVP-3, sample 5—BPA.DM-NVP-4 and sample 6—BPA.DM-NVP-5.

Figure 15 shows the samples after the combustion test. Sample 1 (without talc) burned the fastest. The other materials burned similarly. No large effect of talc particle size on the burning distance was observed, but in comparison with the pure copolymer, the samples with talc maintained their shape. The flammability tests of the materials show clearly that the addition of talc affects their burning behaviour. The BPA.DM-NVP-0 copolymer (without the additives) burned fastest and most intensively. The addition of talc caused a delay in the combustion process and reduced the burning temperature. This effect is best seen the samples with a larger talc particle size.

## 4. Conclusions

The UV-polymerization method was successfully used for the synthesis of crosslinked composites based on bisphenol A glycerolate di-methacrylate (BPA.DM), and N-vinyl-2-pyrrolidone (NVP). Talc with different particle sizes (≤8, 15, 35, 50, 250, 500 and 710 µm) was used as a filler. The ≤500 and ≤710 µm particles of talc were too heavy to achieve a uniform filler distribution in the composite.

Analyzing the change of stress values in relation to the cross-sectional area of the tested samples it is clear that larger particles did not bind with the polymer matrix (BPA.DM-NVP-5). The profilometric tests showed that the surface roughness of the samples correlates directly with the size of the talc particles.

In terms of the physicochemical properties of the composites, it was noted that the particle size did not practically affect the swelling of the composites in water. The tensile modulus values of the samples decrease when larger talc particles are used. Under the bending conditions, the highest values were obtained for the composites containing talc particles of ≤15 µm and ≤35 µm.

The size of the talc particles influences the burning behaviour of the composites. The addition of talc delayed the combustion process and decreased the combustion temperature by more than 10%. The biggest difference in relation to the polymer matrix was obtained for the composites with particles of ≤250 µm in size.

The research proved that the addition of talc can affect the physicochemical parameters of the polymer composites positively while using particles of different sizes, and it is possible to intensify or weaken a given feature.

## Figures and Tables

**Table 1:** Experimental parameters of the synthesis.

Sample No.	Sample Name	BPA.D (g)	NVP (g)	IQ (g)	Talc (g)	Talc, Particle Size, ≤ (µm)
1	BPA.DM-NVP-0	8.4	3.6	0.24	2.4	0
2	BPA.DM-NVP-1					8
3	BPA.DM-NVP-2					15
4	BPA.DM-NVP-3					35
5	BPA.DM-NVP-4					50
6	BPA.DM-NVP-5					250
7	BPA.DM-NVP-6					500
8	BPA.DM-NVP-7					710

**Table 2:** Swelling studies results.

Composite	Swelling Coefficients, B/%				
	Dry	15 min	60 min	720 min	2 Weeks
BPA.DM-NVP-0	0.2377	0.2410	0.2411	0.2414	0.2503 (5.3%)
BPA.DM-NVP-1	0.2708	0.2730	0.2717	0.2739	0.2829 (4.5%)
BPA.DM-NVP-2	0.2649	0.2667	0.2675	0.2691	0.2777 (4.8%)
BPA.DM-NVP-3	0.2686	0.2703	0.2714	0.2731	0.2800 (4.2%)
BPA.DM-NVP-4	0.2648	0.2668	0.2666	0.2684	0.2782 (5.1%)
BPA.DM-NVP-5	0.2800	0.2820	0.2819	0.2837	0.2930 (4.6%)

**Table 3:** Data of mechanical properties.

STRENGTH Testing					
Sample	Young’s Modulus E (MPa)	Tensile Strength σ_m_ (MPa)	Elongation ε_m_ (%)	Sample Width b (mm)	Sample Height h (mm)
BPA.DM-NVP-0	2563.63 ± 13.41	34.54 ± 5.23	1.61 ± 0.59	9.24	1.96
BPA.DM-NVP-1	3622.53 ± 9.55	31.56 ± 3.75	0.93 ± 0.11	8.73	1.83
BPA.DM-NVP-2	3516.43 ± 11.33	38.25 ± 4.,12	1.43 ± 0.98	9.26	1.99
BPA.DM-NVP-3	3471.36 ± 12,56	36.36 ± 4.76	1.39 ± 0.85	9.29	1.96
BPA.DM-NVP-4	3218.21 ± 10.32	39.21 ± 3.96	1.79 ± 0.78	9.24	1.99
BPA.DM-NVP-5	2987.74 ± 12.12	19.76 ± 4.80	0.68 ± 0.56	9.33	1.89
**BENDING Testing**					
**Sample**	**Young’s Modulus** **E_f_ (MPa)**	**Flexural Strength** **σ_fM_ (MPa)**	**Elongation** **ε_fM_ (%)**	**Sample Heigh** **h (mm)**	**Sample Width** **b (mm)**
BPA.DM-NVP-0	3367.09 ± 18.45	51.17 ± 6.12	1.43 ± 0.40	1.84	9.04
BPA.DM-NVP-1	5289.93 ± 19.67	68.25 ± 7.34	1.40 ± 0,55	1.93	9.01
BPA.DM-NVP-2	4371.48 ± 17.30	71.81 ± 6.77	2.02 ± 0,39	1.99	9.33
BPA.DM-NVP-3	5018.01 ± 14.02	78.83 ± 4,84	1.90 ± 0,65	1.92	9.51
BPA.DM-NVP-4	4326.10 ± 15.56	69.12 ± 7.11	1.78 ± 0.90	1.95	9.42
BPA.DM-NVP-5	4393.98 ± 18.77	52.15 ± 8.13	1.20 ± 0.55	2.05	8.61

**Table 4:** Data from the thermographic camera *.

Sample	T_min1_ (°C)	T_max1_ (°C)	T_min2_ (°C)	T_max2_ (°C)
BPA.DM-NVP-0	22.6	248.0	25.5	245.9
BPA.DM-NVP-1	20.6	240.7	23.4	239.0
BPA.DM-NVP-2	22.5	242.1	24.8	241.4
BPA.DM-NVP-3	22.7	240.9	25.1	236.3
BPA.DM-NVP-4	22.6	245.9	24.8	241.5
BPA.DM-NVP-5	22.1	222.9	24.0	212.6

* measurement accuracy of 3%, with the thermal resolution of the NEDT transmitter ±2 °C.

## Data Availability

Not applicable.
